# Penile Girth Injection Complications: A Case Report

**DOI:** 10.1016/j.esxm.2021.100445

**Published:** 2021-10-22

**Authors:** Alden Baird Bryce, Nick Robertson, A Broderick Gregory

**Affiliations:** 1 Mayo Clinic Jacksonville, Jacksonville, FL; 2 Medical College of Georgia, Augusta, Georgia

**Keywords:** Penis, Hyaluronic Acid, Dermatitis, Girth Injection

## Abstract

**Introduction:**

Penile enhancement procedures are becoming more common in men looking to achieve a more desirable penile aesthetic. We describe a case of post-procedural dermatitis after receiving penile girth enhancement injections in an adult male and discuss management of penile girth injection side effects.

**Materials and Methods:**

We review and present our case alongside a discussion of girth injection complications.

**Results:**

Patient's exam and symptoms improved after treatment.

**Conclusion:**

Post-procedural complications after penile girth injections seem to be under-reported and unfortunately can result in severe deformity and dysfunction of the penis.

**Baird Bryce A, Robertson N, Broderick Gregory A. Penile Girth Injection Complications: A Case Report. Sex Med 2021;9:100445.**

## INTRODUCTION

Penile augmentation procedures can result in significant complications. Penile girth enhancement has become a more common procedure sought after by men desiring penile enhancement. Dermal injections and fillers have not been approved by the Food and Drug Administration (FDA) in the United States; however, many patients are exploring girth enhancement options. Our case represents a potential dermatologic complication of penile girth enhancement procedures.

## CASE REPORT

A 56-year old male presented to the emergency department (ED) for concerns of penile pain and swelling over a period of two weeks. The patient had hyaluronic acid injections for penile girth bulking at an outside men's health clinic 2 weeks prior to his emergency department presentation. The man stated he had been taking prednisone since the injection procedure as well as a 10-day course of doxycycline. The patient had previously had bulking injections 9 years ago at the same clinic without significant reported complications.

**Figure 1:**
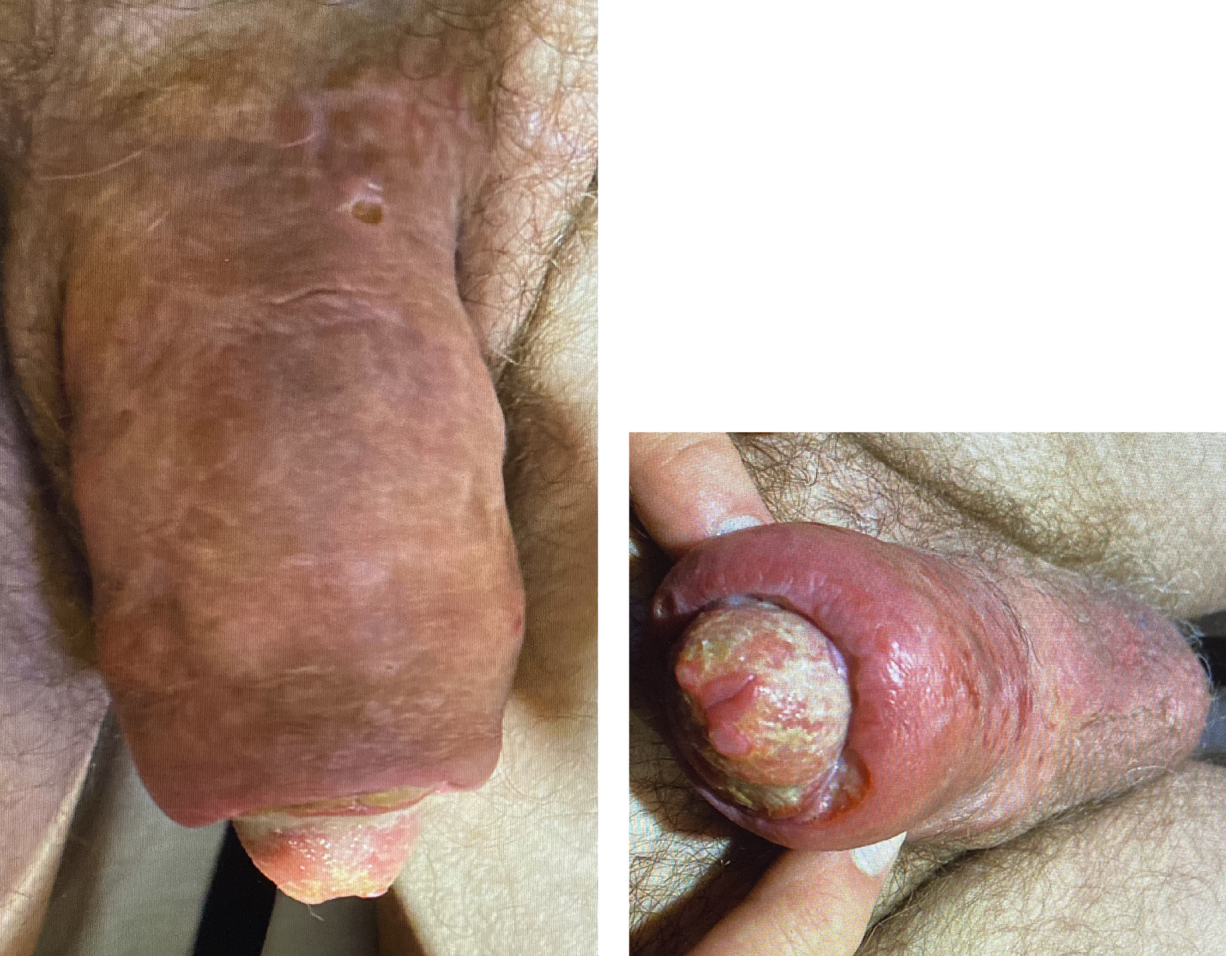
A, Shaft skin changes. B, Glans skin changes.

On physical exam, the patient appeared non-toxic with normal vital signs; he was alert and had a complaint of moderate penile pain which increased with palpation. The patient's penis was swollen and erythematous (Figure 1A). Skin breakdown was evident on the ventral shaft and dorsal base of the penis (Figure 1A and 1B). A yellow exudate was noted at the glans (Figure 1B). Testicular exam was significant for decreased testicular volume but was otherwise within normal limits. On further questioning, the patient admitted to supplemental testosterone use.

Upon the patient's presentation to the emergency department, a CT Pelvis with and without contrast was obtained which showed diffuse heterogeneously dense penile skin thickening with no definitive calcifications. The CT scan combined with physical exam revealed no concern for gas in superficial or deep tissues and showed no evidence of Fournier's gangrene.

At this point, the patient was offered skin biopsy versus skin scrapings to determine a histologic diagnosis. We explained that this diagnostic work-up could provide more specific information about a diagnosis to better treat the pathology. The patient was not interested in interventional diagnostics or therapies given the fact he was enduring a complication of a procedure at that time. We explained that if symptoms did not improve we would need to proceed with a pathologic and/or histologic analysis, and the patient was agreeable to this.

The patient was discharged from the ED with a 14-day course of amoxicillin and/or clavulanate to cover for a presumed infectious cause of his penile swelling. The patient was seen by dermatology two days after discharge. Herpes simplex virus (HSV) and Varicella zoster virus (VZV) polymerase chain reaction (PCR) swabs were obtained as well as bacterial cultures which were all negative. The patient was diagnosed with dermatitis and penile edema secondary to subcutaneous injection of hyaluronic acid and was prescribed topical hydrocortisone 2.5% for as needed use and daily mupirocin ointment. He was followed by Urology 1 week later in clinic with complaints of continued penile tenderness and erythema despite daily use of the topical hydrocortisone, amoxicillin and/or clavulanate, and mupirocin. Approximately one week later, the patient had improvement of symptoms and was recommended continued antibiotic ointment for a small residual ulceration on the dorsal glans. The patient followed with his outside urologist and had resolution of symptoms about one month after his original ED visit.

## DISCUSSION

Penile girth enhancement procedures have been covered in the literature; there are a variety of procedures and injections that can be performed and with that, a variety of complications associated with these interventions. In addition, there is an overall paucity of literature in regard to girth injections, particularly in regard to complications and their medical management.

Penile augmentation procedures can result in significant complications. Dermal injections and fillers have not been approved by the Food and Drug Administration (FDA) in the United States; however, many patients are exploring girth enhancement options. Options include autologous fat, silicone, hyaluronic acid (HA) and collagen injections.^1^ Our case represents a potential dermatologic complication of hyaluronic acid penile girth enhancement procedures along with its management.

Hyaluronic acid-based injections are generally injected via a blunt-tip needle between the superficial or Dartos fascia and the deep or Buck's fascia.^2^ Layers of the penis to consider from superficial to deep include the skin, superficial fascia, deep fascia, and the tunica albuginea that surrounds erectile tissues. These layers and anatomy are important to keep in mind when considering complications. The needle access points are generally at the lateral-most aspects at ‘3 o'clock’ and ‘9 o'clock’ positions. However, our patient had also undergone injections in the glans as has also been described but is less common. There would be a theoretical possibility of glans changes even in patients not undergoing injections directly into the glans. Given the blood supply of the penis, injection elements could possibly travel to the glans via branches of the dorsal penile artery.

In our case, the primary urologist had injected no deeper than deep fascia based on exam and imaging findings. Thus, as far as we could tell, the injection had been administered in the correct plane. Our patient endured swelling, erythema, and hypersensitivity to the hyaluronic acid as complications. Based on the studies we obtained, there was no evidence of infection; however, given the potential extreme consequences of infection including surgical debridement or deep tissue infection resulting in loss of erectile or deeper penile tissues, we proceeded in empiric antibiotic coverage.

There are a variety of complications associated with penile filler injections. Short-term complications include infection, edema, hypersensitivity, erythema, and penile disfigurement.^1^ These short-term complications are noted to occur within days or weeks of the injection. In our case, the patient had an injection approximately 2 weeks prior; although, the patient did have his original injection about a decade earlier. In our case, we could establish a clear short-term source of complication. Fortunately, our patient did not have evidence of fluid collection or gas on CT scan. CT scans are likely superior to magnetic resonance imaging (MRI) in terms of looking for deeper infections that involve gas-forming bacteria; CT scans are much more rapid and have good sensitivity for detecting subcutaneous emphysema.^1^^,^^3^ In general, infection must be excluded when patients present with erythema, pain, and edema after an injection procedure because of the potential long-term consequences of penile infection.^4^^,^^5^

Specific to hyaluronic acid injections, complications include penile subcutaneous nodules, subcutaneous bleeding, penile edema and infection. The most common of these complications is development of a subcutaneous nodule, with a reported incidence of 2.2%. Development of a subcutaneous nodule typically occurs 2 weeks following HA injection, and can be managed with either surgery or hyaluronidase therapy.^6^

While complications certainly must be considered, one study did show that hyaluronic acid gel injections could be safely and efficaciously administered.^7^ In this series of 41 patients, most patients were satisfied with their girth enhancement and no inflammatory reactions were noted.

On the other hand, a study of 230 men who underwent HA injection therapy showed significant penile edema reported in 21 individuals. All 21 of these patients had the problem of redundant prepuce; the authors recommend preoperative circumcision as a means to prevent penile edema following HA injection therapy.^6^

Artificial fillers would likely have an increased likelihood of inflammatory or allergic reactions compared to autologous fat injections. Both artificial fillers and autologous fat injections could develop bleeding, infection, swelling, or other complications discussed above. The work-up and treatment would largely be similar if not the same for other artificial fillers as well as autologous fat injections aside from the fact that allergic reactions would theoretically be decreased in autologous fat injection.

A systematic review of studies involving penile enhancement procedures found an overall low quality of evidence supporting the use of these procedures.^8^ Additionally, criteria to evaluate and report the efficacy and safety of such procedures as well as patient satisfaction were missing from most studies. Given these limitations of previous studies, complications of penile girth enhancement procedures are likely underreported in the literature.^9^ The potential for serious complications combined with the lack of evidence for patient satisfaction underlie the current lack of support for penile girth enhancement strategies.

## CONCLUSION

Complications following penile girth enhancement procedures are likely under-reported. Complications can range from minor dermatologic reactions to severe deep infections of erectile tissues. Unfortunately, penile and even scrotal deformity can occur in men who previously had normal anatomy leading to lifelong anatomic and even physiologic dysfunction. Urologists should have a basic knowledge of these penile enhancement procedures and the complications which can occur after such procedures.

## STATEMENT OF AUTHORSHIP

Conceptualization, BAB GAB and NR.; Methodology, BAB, GAB and NR.; Investigation, BAB and NR.; Writing – Original Draft, BAB and NR; Writing – BAB, NR and GAB Review & Editing, BAB and NR.; Funding Acquisition, BAB, GAB Resources, BAB, GAB, NR Supervision, GAB
